# Caudal Pneumaticity and Pneumatic Hiatuses in the Sauropod Dinosaurs *Giraffatitan* and *Apatosaurus*

**DOI:** 10.1371/journal.pone.0078213

**Published:** 2013-10-30

**Authors:** Mathew J. Wedel, Michael P. Taylor

**Affiliations:** 1 College of Osteopathic Medicine of the Pacific and College of Podiatric Medicine, Western University of Health Sciences, Pomona, California, United States of America; 2 Department of Earth Sciences, University of Bristol, United Kingdom; University of Pennsylvania, United States of America

## Abstract

Skeletal pneumaticity is found in the presacral vertebrae of most sauropod dinosaurs, but pneumaticity is much less common in the vertebrae of the tail. We describe previously unrecognized pneumatic fossae in the mid-caudal vertebrae of specimens of *Giraffatitan* and *Apatosaurus*. In both taxa, the most distal pneumatic vertebrae are separated from other pneumatic vertebrae by sequences of three to seven apneumatic vertebrae. Caudal pneumaticity is not prominent in most individuals of either of these taxa, and its unpredictable development means that it may be more widespread than previously recognised within Sauropoda and elsewhere in Saurischia. The erratic patterns of caudal pneumatization in *Giraffatitan* and *Apatosaurus*, including the pneumatic hiatuses, show that pneumatic diverticula were more broadly distributed in the bodies of the living animals than are their traces in the skeleton. Together with recently published evidence of cryptic diverticula—those that leave few or no skeletal traces—in basal sauropodomorphs and in pterosaurs, this is further evidence that pneumatic diverticula were widespread in ornithodirans, both across phylogeny and throughout anatomy.

## Introduction

Postcranial skeletal pneumaticity (PSP) is the modification of the postcranial skeleton by pneumatic diverticula of the respiratory system. It is widespread in saurischian dinosaurs including birds, other theropods, and sauropods, and it is also present in pterosaurs. PSP in archosaurs is of interest as a morphogenetic system and source of phylogenetic information [1]–[3], for its effect in lightening the skeleton [4]–[8], as the skeletal footprint of the lungs and air sacs [9]–[17], and as the osteological correlate of a system of pneumatic diverticula, which developed from the lungs and air sacs and may have had important non-respiratory functions [18], [19]. The extent of PSP varied greatly among sauropod taxa, among individuals and among regions of the skeleton. Cervical vertebrae are pneumatic in basal eusauropods; cervical, dorsal and sacral vertebrae are pneumatic in mamenchisaurids and most neosauropods; and all of these plus caudal vertebrae are extensively pneumatic in diplodocines and in some titanosaurians [1], [4], [12], [20]. Cervical and dorsal ribs are pneumatic in many, maybe most, titanosauriforms (e.g., [21]: p. 239; [22]: p. 52) and some diplodocids (e.g., [23]: figs. 9–10; 24: p. 212; [25]: p. 534). Pectoral girdle elements are pneumatic in some derived titanosaurs [20], and pneumatization of pelvic girdle elements apparently evolved independently in rebbachisaurid diplodocoids [26]–[27] and somphospondylan macronarians ([20], [28]: p. 233). Most of the elements listed above are also pneumatized in at least some pterosaurs [7], non-avian theropods [13], [15], and birds [6], [13], [14], [29], although caudal pneumaticity has not yet been demonstrated in pterosaurs, and ischial pneumaticity is not yet known in non-avian theropods [27]. The acquisition of PSP in parallel in so many ornithodiran lineages suggests that a diverticular lung and air sac system may be primitive for Ornithodira as a whole [12], [15]–[17].

To date, caudal pneumaticity has received less attention than pneumaticity in other parts of the skeleton (but see [30]), but it is of particular interest because of its possible independent origins and parallel evolution in diplodocoids and macronarians. Here we describe complex patterns of caudal pneumaticity in *Giraffatitan brancai* (formerly assigned to the genus *Brachiosaurus*; see [31]) and *Apatosaurus*, and discuss the functional and phylogenetic implications.

### Institutional Abbreviations

**AMNH**, American Museum of Natural History, New York City, New York, USA; **CM**, Carnegie Museum of Natural History, Pittsburgh, Pennsylvania, USA; **DMNH**, Denver Museum of Natural History, Denver, Colorado, USA; **FMNH**, Field Museum of Natural History, Chicago, Illinois, USA; **HMN**, Humbolt Museum für Naturkunde, Berlin, Germany; **KLR**, Henan Geological Museum, Zhengzhou, China; **LACM**, Natural History Museum of Los Angeles County, Los Angeles, California, USA; **MAL**, Malawi Department of Antiquities Collection, Lilongwe and Nguludi, Malawi; **MB.R.**, Museum für Naturkunde Berlin, Berlin, Germany; **MCS**, Museo de Cinco Saltos, Río Negro Province, Argentina; **MCT**, Collection of the Earth Science Museum of the National Department of Mineral Production, Río de Janeiro; **MIWG**, Museum of Isle of Wight Geology, Sandown, Isle of Wight, United Kingdom; **ML**, Museu da Lourinhã, Portugal; **MN**, Museu Nacional, Rio de Janeiro, Brazil; **MPCA-Pv**, Colección de Paleovertebrados de la Museum Provincial de Cipolletti “Carlos Ameghino”, Cipolletti, Río Negro Province, Argentina; **MPS**, Museo de Dinosaurios e Paleontología, Salas de los Infantes, Burgos, Spain; **MUCPv**, Museo de Geología y Paleontología de la Universidad Nacional del Comahue, Neuquén, Argentina; **NHM**, Natural History Museum, London, United Kingdom; **NMST**, National Science Museum, Tokyo, Japan; **OMNH**, Oklahoma Museum of Natural History, Norman, Oklahoma, USA; **ONM**, Office National Des Mines, Service Patrimoine Géologique, Tunis, Tunisia; **PVL**, Colección de Paleontología de Vertebrados de la Fundación Instituto Miguel Lillo, Tucumán, Argentina; **UNPSJB**, Universidad Nacional de la Patagonia San Juan Bosco, Comodoro Rivadavia, Argentina; **USNM**, National Museum of Natural History, Smithsonian Institution, Washington, D.C., USA; **WDC**, Wyoming Dinosaur Center, Thermopolis, Wyoming, USA; **YPM**, Yale Peabody Museum, New Haven, Connecticut, USA.

## Results and Discussion

### Overview of pneumatic features

**Figure 1:**
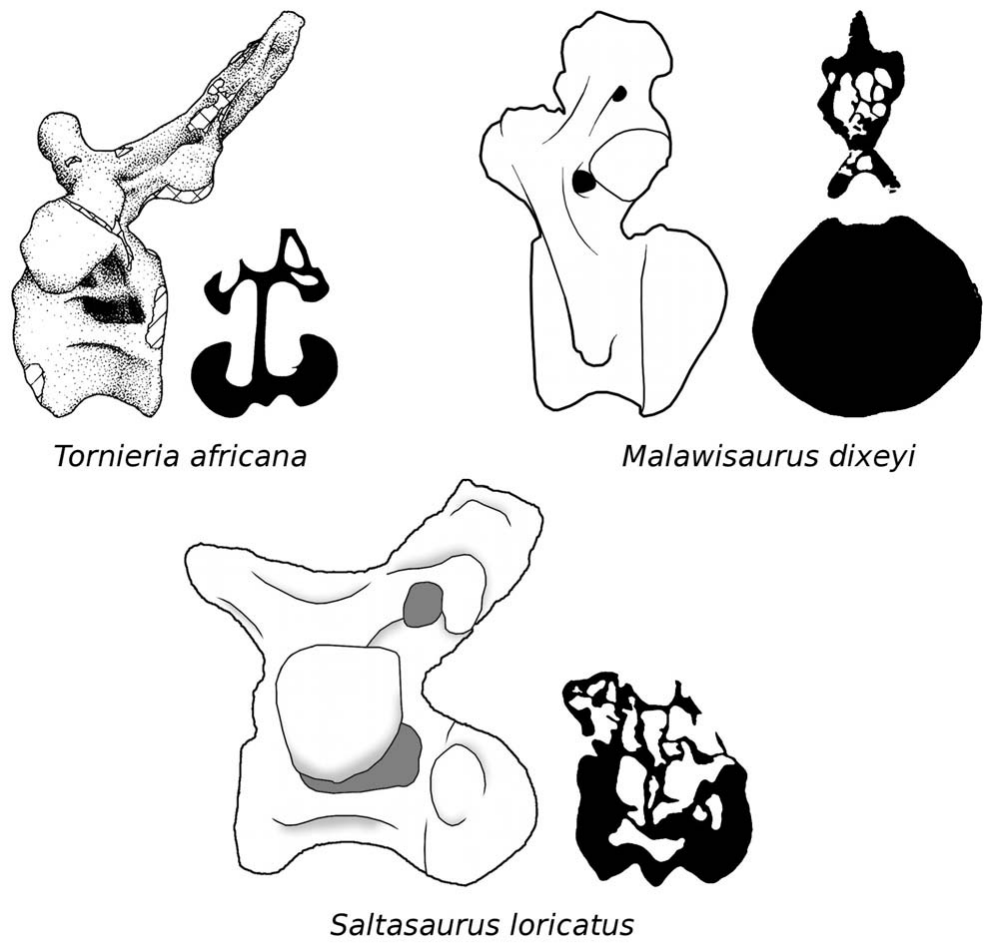
Caudal pneumaticity varies among sauropods. In the diplodocid *Tornieria*, the first 15–20 caudal vertebrae have neural arch laminae and fossae, and lateral pneumatic foramina opening into large internal chambers. Images traced from Remes ([51]: fig. 31 [lateral view]) and Janensch ([72]: fig. 7 [cross-section]); the two views are from different vertebrae. In the basal titanosaurian *Malawisaurus*, caudal pneumaticity is restricted to a handful of proximal caudal vertebrae, in which the neural arches are honeycombed with pneumatic chambers but the vertebral centra are solid. Images traced from Wedel ([12]: fig. 2A [lateral view] and 2C [cross-section]). In the derived titanosaurian *Saltasaurus*, the first 20–25 caudal vertebrae have large external fossae but small external foramina, and both the neural arches and centra are honeycombed with chambers. Images traced from Powell ([59]: plate 53 [lateral view]) and Cerda *et al* [20]: fig. 4F [cross-section]); the two views are from different vertebrae.

The interaction of pneumatic epithelium and bone tissue produces a spectrum of osteological features, including pneumatic tracks, fossae, foramina, and internal chambers of various shapes and sizes [1], [4], [9], [10], [14], [32](**Figure 1**). Not all of these features are diagnostic for pneumaticity in isolation. Pneumatic fossae are particularly problematic: fossae on the surface of vertebrae can be associated with numerous soft tissues, including cartilage, adipose tissue, muscles, and pneumatic diverticula [14]. Although distinctly emarginated and sharply lipped fossae are usually inferred to represent pneumatic invasion [9], apneumatic fossae sometimes have distinct margins and pneumatic fossae sometimes do not [16], [17], [32]. It is worth noting that vertebral fossae are present in numerous basal and pseudosuchian archosarus [16], [17], [33] and in some synapsids (see discussion in [15]: p. 172), and although it is possible that some of these were pneumatic, it is unlikely that all of them were.

**Figure 2:**
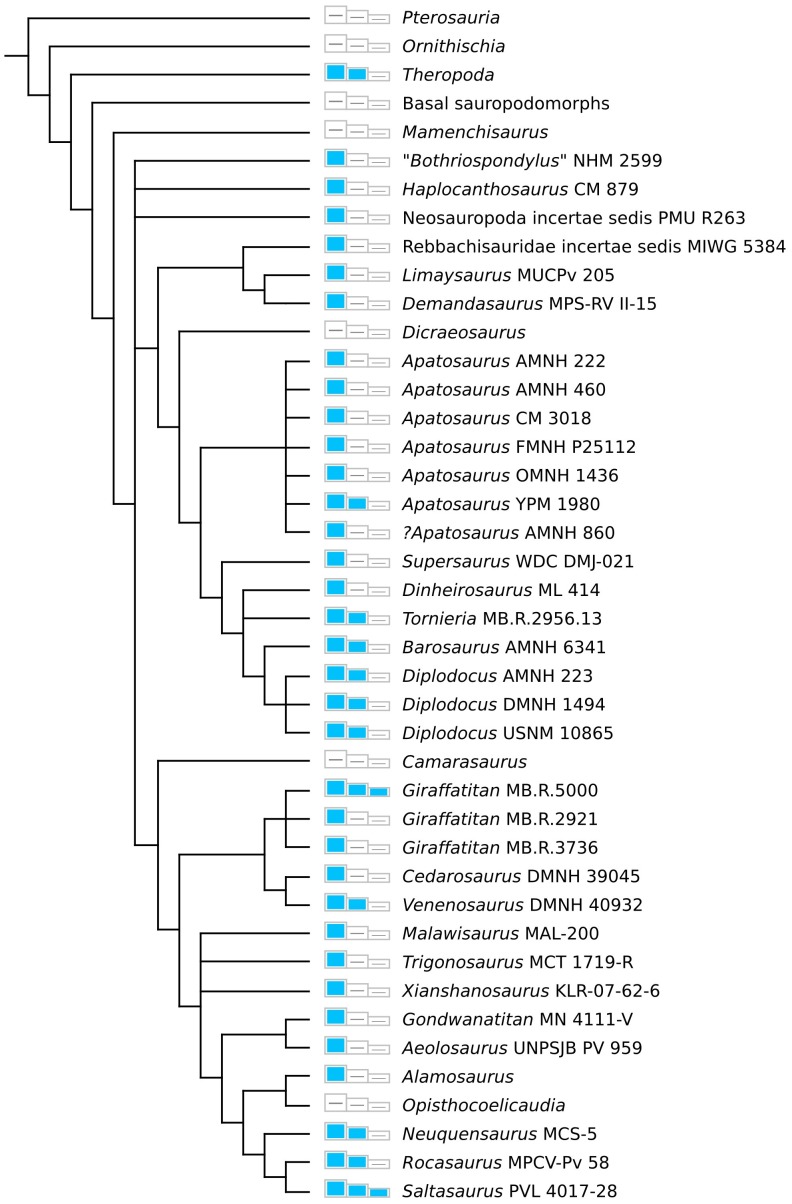
The phylogenetic distribution of caudal pneumaticity in sauropods and other dinosaurs is complex. Boxes represent proximal, middle, and distal caudal vertebrae, arbitrarily defined for sauropods as caudals 1–10, 11–20, and 21 on, respectively; blue boxes indicate that pneumaticity is present in that part of the tail. Pneumaticity data for theropods come from Benson *et al* [15]—note that although Theropoda is collapsed to a single node in this figure, caudal pneumaticity is not primitive for the clade, but evolved independently several times in both non-avian theropods and birds [6], [15], [29]. Data from sauropods come from the sources listed in Table 1. The figure also shows the phylogenetic framework we use in this paper. The phylogenetic framework is drawn from Whitlock [44] for diplodocoids, Mannion *et al* [30] for basal macronarians and *Xianshanosaurus*, Calvo *et al* [96] for most titanosaurs, and Campos *et al* [93] for *Trigonosaurus*. Basal sauropodomorphs are a grade, not a clade, but they are listed together here for convenience since they all lack caudal pneumaticity.

In equivocal cases, the diagnosis of a fossa as pneumatic may be strengthened by the presence of other pneumatic features on the same bone [4]. Unequivocally pneumatic fossae (e.g. those containing pneumatic foramina) often have multiple subfossae [17], [34], which may represent the resorption of adjacent cortical bone by a complex diverticulum that consists of multiple tubes or sacs, such as the complex diverticula of some birds ([11]: fig. 2). Apneumatic fossae usually have no margins or only weakly developed margins; the only strongly emarginated apneumatic fossae are muscle attachments that are easily identified by their location and texture, such as the temporal fossae of the human skull and the muscle attachment fossae on the ilia of birds. PSP in saurischians is typically variable: the presence and form of pneumatic features varies among individuals, serially along the vertebral column, and even on the left and right sides of a single vertebra (e.g., [35]: p. 1552).

Although fossae are less diagnostic for PSP than more invasive foramina and internal chambers, the differences between pneumatic and apneumatic fossae listed above can be used to develop a profile for distinguishing the two ([9], [17]; see also [14]: fig. 12). In descending order of usefulness, pneumatic fossae are expected to (1) occur together with other correlates of PSP, (2) have a scalloped texture or subfossae, (3) occur on bone surfaces not occupied by muscle attachments, or in the same locations as pneumatic foramina in related taxa, and (4) vary in expression among individuals, serially along the axial skeleton, and from left to right in single vertebra. There is no reason to assume that putatively pneumatic fossae were originally occupied by some other soft tissue (e.g., muscle, cartilage, or adipose tissue) which was then replaced by pneumatic diverticula that produced more diagnostic bony traces [17], especially given the mounting evidence that a diverticular lung was present in the ancestral saurischian and possibly in the ancestral ornithodiran [12], [15]–[17]. Nevertheless, it is often difficult to tell which fossae may have been pneumatic, especially in basal taxa or those in which the presence of PSP is unexpected or not well established [16].

### Caudal pneumaticity in Ornithodira

The phylogenetic distribution of caudal pneumaticity in sauropods and in ornithodirans more generally is complex (**Figure 2**). To date, there are no reports of caudal pneumaticity in pterosaurs. There are several possible explanations for this. Although the presence of PSP in pterosaurs has been widely acknowledged since the mid-1800s (e.g., [36]), and although it has received more attention in recent years (e.g., [7], [37]), there has still been less work on pneumaticity in pterosaurs than in sauropods or theropods. So possibly caudal pneumaticity is present in pterosaurs but hasn’t been recognized yet. Caudal vertebrae in pterosaurs are small and at small scale it can be difficult to distinguish pneumatic and vascular foramina, and to tell pneumatic chambers from marrow-filled trabecular bone ([16]: p. 18). It does not help that the pterosaurs with long tails were mostly small-bodied, whereas the large-bodied pterodactyloids had tiny tails. The absolutely small tails of pterosaurs may have created little demand or opportunity for pneumatization, and if any pneumatic traces are present in pterosaur tails they would be difficult to diagnose.

**Figure 3:**
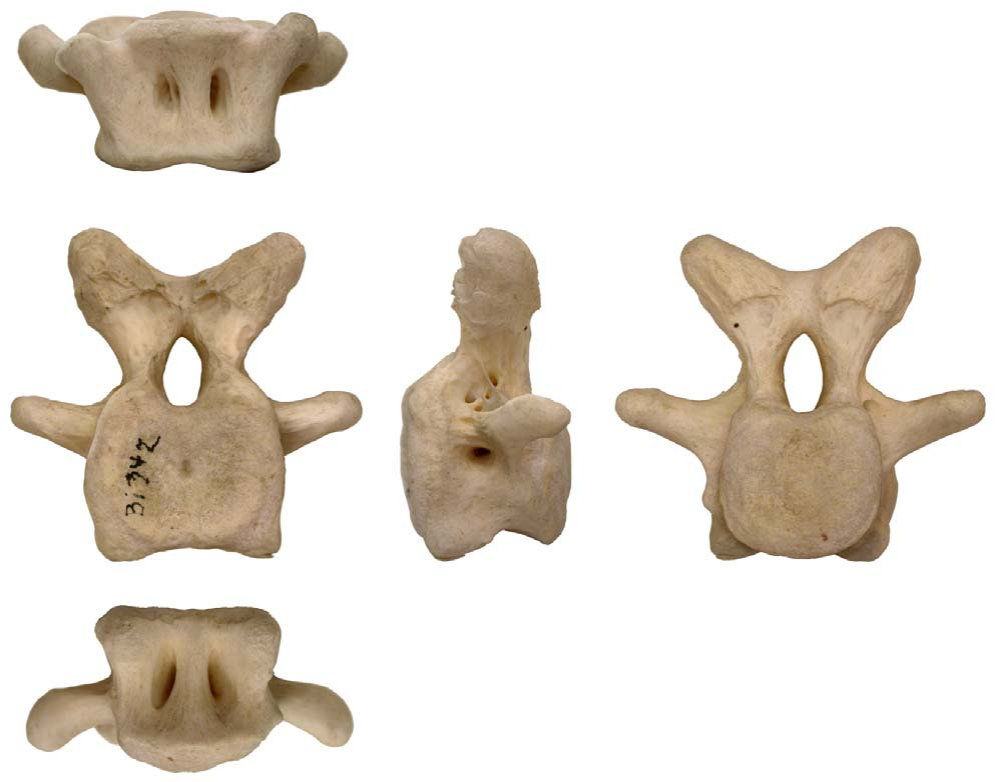
The caudal vertebrae of ostriches are highly pneumatic. This mid-caudal vertebra of an ostrich (*Struthio camelus*), LACM Bj342, is shown in dorsal view (top), anterior, left lateral, and posterior views (middle, left to right), and ventral view (bottom). The vertebra is approximately 5cm wide across the transverse processes. Note the pneumatic foramina on the dorsal, ventral, and lateral sides of the vertebra.

**Figure 4:**
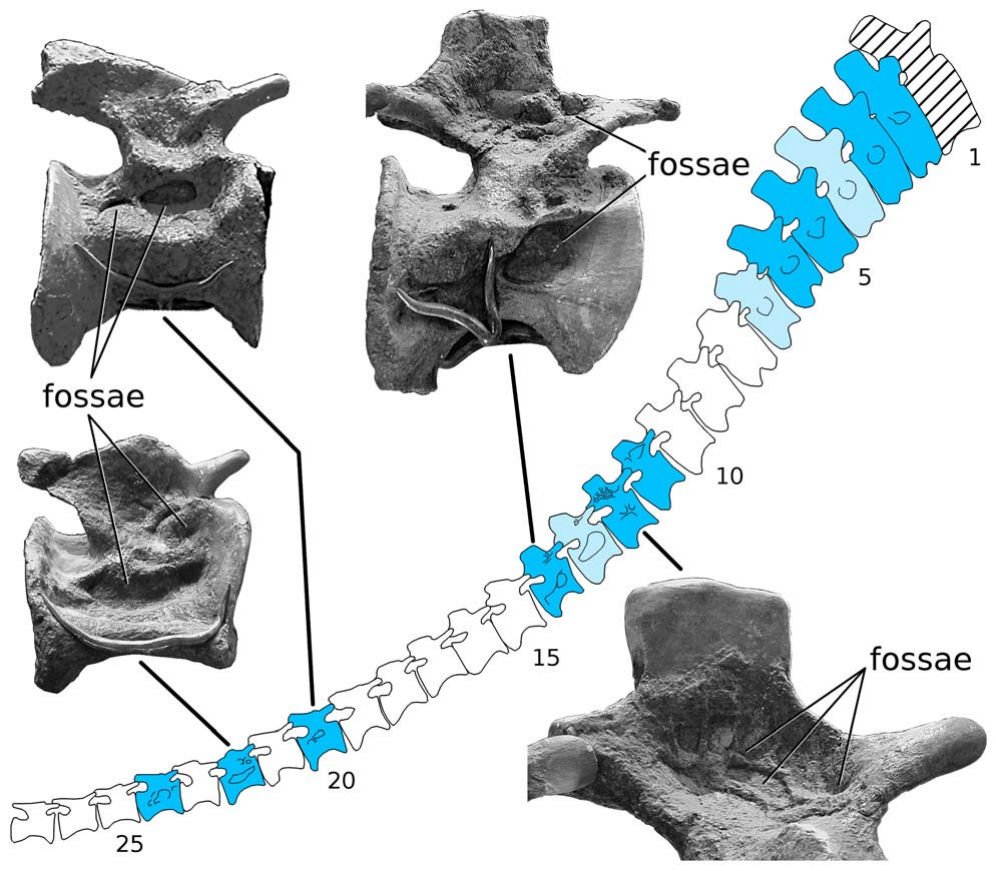
*Giraffatitan brancai* tail MB.R.5000 (‘Fund no’) in right lateral view. Dark blue vertebrae have pneumatic fossae on both sides, light blue vertebrae have pneumatic fossae only on the right side, and white vertebrae have no pneumatic fossae on either side. The first caudal vertebra (hatched) was not recovered and is reconstructed in plaster.

Caudal pneumaticity is uncommon in non-avian theropods. The most comprehensive survey to date is that of Benson *et al* [15], who found caudal pneumaticity in only 12 of the 159 taxa they surveyed. Note, however, that 67 taxa could not be scored, so caudal pneumaticity could be positively ruled out in only half of the sampled taxa (80 out of 159). Only the proximal caudals, if any, are pneumatic in megalosaurids (*Torvosaurus*) and therizinosauroids (*Nothronychus*, *Neimongosaurus*); proximal and middle caudals are pneumatic in some allosauroids (*Aerosteon*, *Megaraptor*, *Carcharodontosaurus*); and proximal, middle, and distal caudals are pneumatic in some—but not all—oviraptorosaurs (*Chirostenotes*, *Citipati*, *Khaan*; see fig. 4, table 4, and appendix S1 in [15]). In contrast, caudal pneumaticity is fairly common in extant birds, at least in medium-to-large-bodied taxa: O'Connor ([6]: table 2) found caudal pneumaticity in at least some members of 6 out of 10 higher-level clades (mostly corresponding to traditional Linnean orders). In addition to the volant taxa surveyed by O'Connor [6], the large ratites (ostriches, emus, cassowaries, and rheas) all have pneumatic caudals (pers. obs., **Figure 3**).

In general, caudal pneumaticity is common in neosauropods and rare or absent in non-neosauropod sauropodomorphs (**Table 1**). A proximal caudal of ‘*Bothriospondylus madagascarensis*’, NHM 2599, has fossae on the lateral sides of the centrum, but lacks large pneumatic foramina or internal pneumatic chambers [38]. The phylogenetic position of the ‘*B. madagascarensis*’ material is uncertain and it may not all pertain to the same taxon [38]. Mannion [38] suggested that it might best be regarded as a non-neosauropod eusauropod, at least until more complete and diagnostic material comes to light. If NHM 2599 does belong to a eusauropod, it is probably the best documented case of caudal pneumaticity in a non-neosauropod sauropodomorph. Caudal pneumaticity has not been reported in the Mamenchisauridae, a clade which otherwise shows some derived pneumatic features, including complex pneumatic chambers in the cervical vertebrae [39].

**Table 1:** Most posterior pneumatic caudal vertebra in several sauropods.

Clade	Genus	Specimen	Caudal #^a^	Reference
Eusauropoda	‘*Bothriospondylus*’	NHM 2599	proximal	[38]
Neosauropoda	*Haplocanthosaurus*	CM 879	1	[12]
	Neosauropoda incertae sedis	PMU R263	proximal	[87]
Rebbachisauridae^b^	*Demandasaurus*	MPS-RV II-15	proximal	[47]
	*Limaysaurus*	MUCPv 205	proximal	[46]: fig. 3
	*Tataouinea*	ONM DT 1-36	proximal	[27]
	Rebbachisauridae incertae sedis	MIWG 5384	proximal	[46]: figs. 1-2
	Rebbachisauridae incertae sedis	NHM R36636	proximal	[88]
Diplodocidae	*Apatosaurus*	AMNH 222	proximal	[74]
		AMNH 460	5	[53]: 188
		CM 3018	3	pers. obs.
		FMNH P25112	5	[53]: 189
		OMNH 1436	proximal	pers. obs.
		YPM 1980	13	pers. obs.
	?*Apatosaurus*	AMNH 860	proximal	pers. obs.
	*Dinheirosaurus*	ML 414	proximal	[89]
	*Supersaurus*	WDC DMJ-021	proximal	[25]
	*Barosaurus*	AMNH 6341	14	pers. obs.
		YPM 429	17 or 19	[50], [90]
	*Diplodocus*	AMNH 223	18	[48]
		DMNH 1494	16	pers. obs.
		USNM 10865	19	[65]
	*Tornieria*	MB.R.2956.13	middle	[51]
Brachiosauridae	*Giraffatitan*	MB.R.2181	24	pers. obs.
		MB.R.2921	2	pers. obs.
		MB.R.3736	2	pers. obs.
		‘Fund G1’	2	[57]
	*Cedarosaurus*	DMNH 39045	proximal	[55]
	*Venenosaurus*	DMNH 40932	middle	[56]
Titanosauria	*Malawisaurus*	MAL-200	proximal	[12]
	*Gondwanatitan*	MN 4111-V	?3	[91]
	*Aeolosaurus*	UNPSJB PV 959	proximal	[92]
	*Trigonosaurus*	MCT 1719-R	?2	[93]
	*Xianshanosaurus*	KLR-07-62-06	proximal	[94]
	*Alamosaurus*	(unspecified)	proximal	[95]
	*Rocasaurus*	MPCV-Pv 58	middle	[20]
	*Neuquensaurus*	MCS-5	middle	[20]
	*Saltasaurus*	PVL 4017-28	distal	[20]

a In several specimens the precise serial position is unknown; in these cases the approximate location in the tail is given as proximal (caudals 1–10), middle (caudals 11–20), or distal (caudals 21 and higher).

b For more discussion on caudal pneumaticity in rebbachisaurids, see [46] and [88].

The first caudal vertebra of *Haplocanthosaurus* CM 879, has pneumatic fossae on both the centrum and the neural arch ([40]: plate 2; [12]: figs. 7 and 9). The phylogenetic position of *Haplocanthosaurus* is uncertain; it has been recovered as a basal diplodocoid [41], a basal macronarian [22], [42], and a non-neosauropod close to the origin of Neosauropoda [43] in different analyses, although recent analyses tend to support a position within Diplodocoidea [25], [44]. Here we regard it as a neosauropod of uncertain affinities (**Figure 2**); moving it into either Diplodocoidea or Macronaria would have no great effect on the phylogenetic distribution of caudal pneumaticity in sauropods. In more derived diplodocoids, caudal pneumaticity is present in rebbachisaurids and diplodocids but apparently absent in dicraeosaurids (see [45]). In rebbachisaurids the neural arches and transverse processes of the proximal caudals often have pronounced laminae and deep, irregular fossae characteristic of pneumaticity ([46]: figs. 1-3; [47]), and pneumatic foramina leading to large internal chambers are present in at least the proximal caudals of the rebbachisaurid *Tataouinea* (the middle and distal caudals are as yet unknown) [27]. The same is true in diplodocids, and in diplodocines such as *Diplodocus*, *Barosaurus*, and *Tornieria*, these pneumatic foramina persist down to caudal 15 or 20 (48: fig. 13; [49]: p. 35 and plate 9; [50]: p. 54 and fig. 2.6; [51]: fig. 3). Although some authors have reported pneumatic features in the most proximal caudal vertebrae of *Apatosaurus* (e.g., [52], [53]), pneumatic features have not previously been observed further back than the fifth caudal vertebra; below we report isolated pneumatic fossae more distally in the tail.

Pneumaticity is absent in the caudal vertebrae of *Camarasaurus* (see [54]: plates 74–77) but caudal pneumaticity is otherwise prevalent in Macronaria. Pneumatic fossae have been reported in the caudals of the brachiosaurids *Cedarosaurus* [55] and *Venenosaurus* [56], and Janensch [57] briefly mentioned fossae in proximal caudal vertebrae in three specimens of *Giraffatitan* (discussed in more detail below). Below, we describe additional pneumatic fossae distributed unevenly through the tail in another specimen of *Giraffatitan*. Caudal pneumaticity is also widespread in Titanosauria ([30]; **Table 1**), with *Opisthocoelicaudia* being one of the few titanosaurs that appears to lack caudal pneumaticity (see [58]: plates 4–5). Caudal pneumaticity reached its apex among sauropods in the saltasaurines *Rocasaurus*, *Neuquensaurus*, and *Saltasaurus*, as did appendicular pneumaticity [20]. Known saltasaurines are uniformly small, with femur lengths well under one meter [59]–[61]—compare to femur lengths of 1–1.2 meters in dicraeosaurids and 1.5–2.0 meters in most other neosauropods ([62]: table 1). It is not yet clear why PSP, which is suspected to have been a key innovation in facilitating the evolution of large body size in sauropods [63], achieved its maximum expression in these small-bodied taxa.

### Caudal pneumaticity in *Giraffatitan*

Caudal vertebrae of *Giraffatitan* personally examined by us in this study are listed in **Table 2**, and described below.

**Table 2:** Caudal vertebrae of *Giraffatitan* in the Museum für Naturkunde Berlin personally examined by us in this study.

Specimen	Field #	Caudal #	Pneumatic?	Fossae and Foramina
MB.R.5000^a^	no	2–51	Yes	scattered fossae to Ca24
MB.R.2921	Aa	1–18	Yes	fossae only on Ca2
MB.R.3736	D	1–31	Yes	fossae only on Ca2
MB.R.3748	dd	middle caudal	No	
MB.R.3786	St 10	middle caudal	No	
MB.R.3787	St 274	middle caudal	No	
MB.R.4029^b^	P	proximal centrum	No	
uncatalogued	G1	proximal series	Yes	fossae reported in Ca2 by [57] c
MB.R.3450^d^	?	proximal centrum	No	
MB.R.4030	?	middle caudal	No	
MB.R.4038	?	proximal centrum	No	
MB.R.4041	?	proximal centrum	No	neurovascular foramina only

a MB.R.5000 (‘Fund no’) is incorporated into the famous mounted skeleton with MB.R.2181.

b MB.R.4029 may pertain to *Janenschia* rather than *Giraffatitan*, but as it shows no evidence of pneumaticity it does affect our findings.

c We were unable to locate the pneumatic vertebra from site G1 reported by [57], although we did examine several apneumatic vertebrae from the site. We were also unable to locate the vertebrae from site Y.

d MB.R.3450 might be part of the caudal series from site G1.

#### MB.R.5000 (‘Fund no’, Figures 4 and 5)

The mounted skeleton of *Giraffatitan brancai* at the Humboldt Museum für Naturkunde Berlin consists primarily of elements of the paralectotype, MB.R.2181 (formerly cataloged as HMN SII), but missing parts of the skeleton were provided from the remains of other similarly sized individuals [64]. The tail of the mounted skeleton, MB.R.5000 (formerly HMN ‘Fund no’), consists of the second to fifty-first caudal vertebrae, “not articulated, with the exception of a few at the end, but altogether relatively in sequence” ([57]: p. 64, plate IV; **Figure 6**). The first caudal vertebra was not recovered, and it is modeled in plaster in the mounted skeleton. The preserved caudals are discussed in groups of serially adjacent vertebrae based on pneumatic characters.

**Figure 5:**
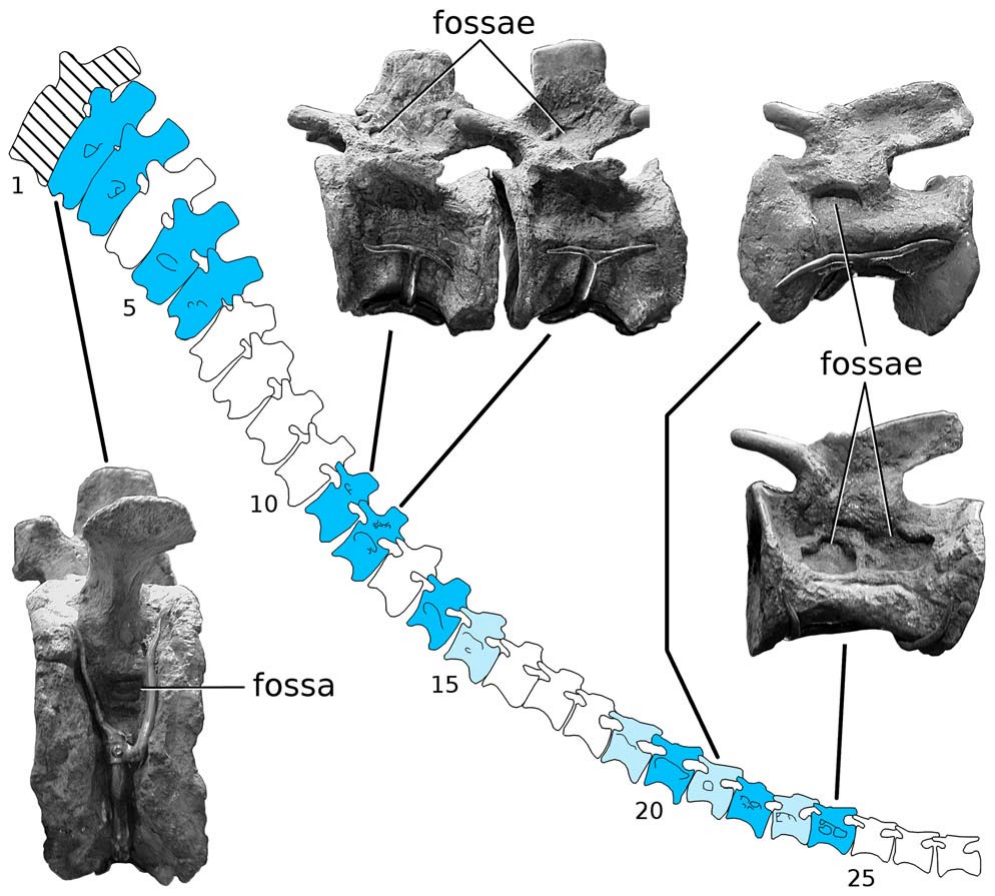
*Giraffatitan brancai* tail MB.R.5000 (‘Fund no’) in left lateral view. Shading conventions follow Figure 4, with light blue vertebrae having pneumatic fossae only the left side.

**Figure 6:**
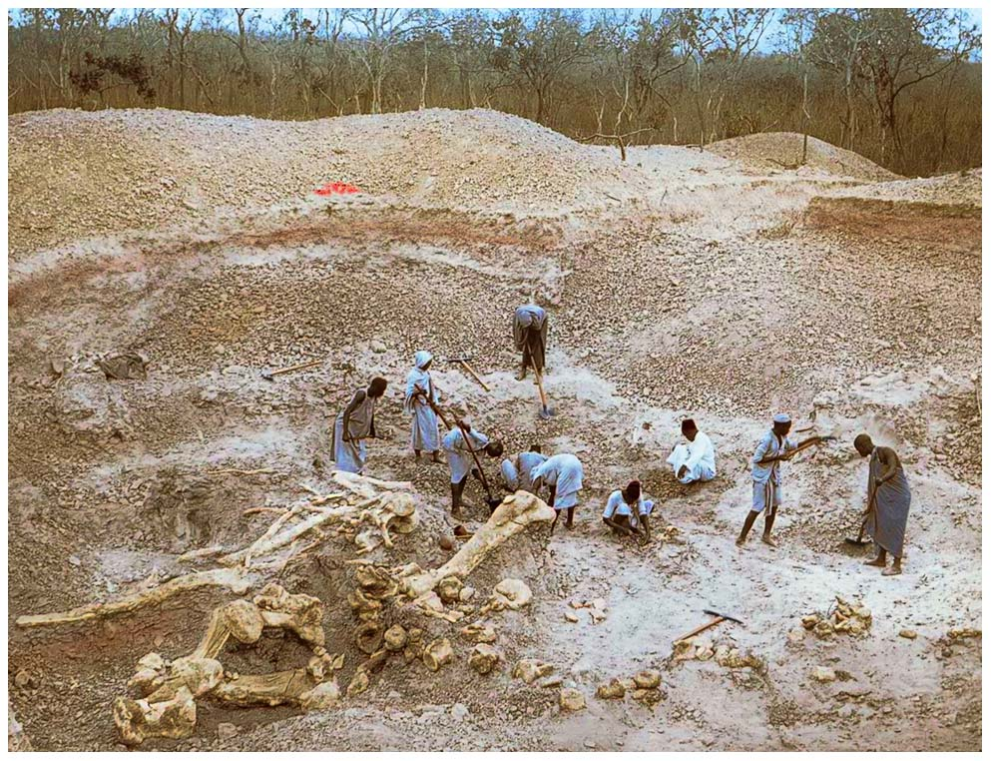
The ‘Fund no’ quarry at Tendaguru preserved a tail of *Giraffatitan* with the vertebrae roughly in order. The series of caudal vertebrae catalogued as MB.R.5000 and incorporated in the famous mounted skeleton of *Giraffatitan* are visible near the bottom of the photo. The photo appears courtesy of the Museum für Naturkunde Berlin.

#### MB.R.5000 (‘Fund no’): Caudal vertebrae 2–7

All of these vertebrae have fossae on the right side of the centrum, and all but Ca4 and Ca7 also on the left. The fossae of these vertebrae are all located ventral to the transverse processes on the dorsolateral faces of the centra. Some of the fossae are multipartite; that is, divided into subfossae by bony septa. Fossae are absent from the neural arches and spines. Caudals 4 and 7 have fossae only on the right side of the centrum: similar asymmetry in the expression of pneumatic fossae is present in the sacrum of the CM 879 specimen of *Haplocanthosaurus* [12].

#### MB.R.5000 (‘Fund no’): Caudal vertebrae 8–10

Although these vertebrae present a series of intermediate forms relative to the vertebrae anterior and posterior to them, and all are deeply waisted, they have no apparent pneumatic features on their centra, neural arches, or neural spines. As there are obvious traces of pneumaticity in caudal vertebrae 11–15 (see below), pneumatic diverticula must have passed by these vertebrae and may even have been in contact with the bone, but they left no macroscopic traces. It is possible that correlates of PSP might be found in the bone microtexture or histology of these vertebrae, but such correlates have not been identified to date in any vertebrae so resolution of this question must wait. This block of three vertebrae is bounded anteriorly and posteriorly by pneumatic vertebrae and thus constitutes a pneumatic hiatus [11], [12]; the implications of this hiatus are explored below.

#### MB.R.5000 (‘Fund no’): Caudal vertebrae 11–15

All of these vertebrae have pneumatic fossae, and the distribution and morphology of these fossae is considerably more complex than in caudals 2–7. The most obvious difference between these ranges is that those in the posterior range have pneumatic fossae on both the centrum and neural arch, whereas more anteriorly fossae are present only on the centrum. Caudal vertebra 11 has fossae on both sides of the neural arch, and these fossae are weakly subdivided by bony septa. No fossae are apparent on either side of the centrum. Caudal vertebra 12 has the most complex pneumatic features of any vertebra in the entire tail, with multipartite fossae on both sides of the centrum and both sides of the neural arch. Caudal vertebra 13 has a very large fossa on the right side of the centrum, which in its size and form approximates the large pneumatic fossae or “pleurocoels” in the dorsal vertebrae of more basal taxa like *Haplocanthosaurus*. A small subdivided fossa is also present on the right side of the neural spine. Pneumatic features are absent from both the centrum and neural arch on the left side. Caudal 13 is therefore similar to caudals 4 and 7 in having pneumatic features present only on the right side. Caudal 14 has large pneumatic fossae on both sides of the centrum, and a smaller multipartite fossa on the right side of the neural arch. Caudal 15 has a pair of pneumatic fossae on the left side of the centrum, but no fossae on the neural arch or anywhere on the right side of the vertebra. This is the first vertebra in the series in which PSP is present only on the left side; all of the previous vertebrae that are unilaterally apneumatic (caudals 4, 7 and 13) have their fossae on the right side.

#### MB.R.5000 (‘Fund no’): Caudal vertebrae 16–18

These three vertebrae, like caudals 8–10, are deeply waisted but lack distinct fossae. They constitute a second bilateral pneumatic hiatus.

#### MB.R.5000 (‘Fund no’): Caudal vertebrae 19–24

These six vertebrae again present a complex suite of pneumatic features. Caudals 19, 21, and 23 have pneumatic fossae only on the left side, like caudal 15, whereas caudals 20, 22, and 24 have pneumatic fossae on both sides of the centrum. Caudal 22 has a multipartite fossa on the right side, on the border between the centrum and neural arch; fossae are otherwise absent from the neural arches and spines of all six vertebrae. In contrast, pneumatic fossae on the centra of these six vertebrae are better defined than in almost all of the preceding vertebrae, with the fossae of caudals 20, 22, and 24 being particularly large, deep, and well subdivided.

#### MB.R.5000 (‘Fund no’): Caudal vertebrae 25–51

No obvious pneumatic features are present on any of these vertebrae. The vertebrae that make up the last 26 cm of the tail (i.e. from caudal 52 on) were not recovered and are reconstructed in plaster in the mounted skeleton ([64]: p. 98). We assume that the missing vertebrae were also apneumatic, based on the absence of pneumaticity in the preceding 27 vertebrae and in the distal tails of all other known non-avian saurischians.

#### MB.R.2921 (‘Fund Aa’, Figure 7)

MB.R.2921 (‘Fund Aa’) consists of the first 18 caudal vertebrae and their chevrons, found in an articulated sequence behind the last sacral vertebra ([57]: p. 60). Regarding possible pneumatic features, Janensch ([57]: p. 61) wrote, “Pleurocentral excavations are absent; only under the root of the transverse process of the second is an elongated, about four centimeter long depression clearly developed, particularly on the right.” We have confirmed that small fossae are present on both sides of the centrum in the second caudal, and that they are absent from the first caudal. These fossae are similar to those found in the first pneumatic block (caudals 2–7) of MB.R.5000 (‘Fund no’; see above). Fossae are absent on the neural arch of the second caudal, and in all the other caudal vertebrae that make up the specimen. The first caudal vertebra of MB.R.2921 (‘Fund Aa’) therefore constitutes another (short) pneumatic hiatus.

**Figure 7:**
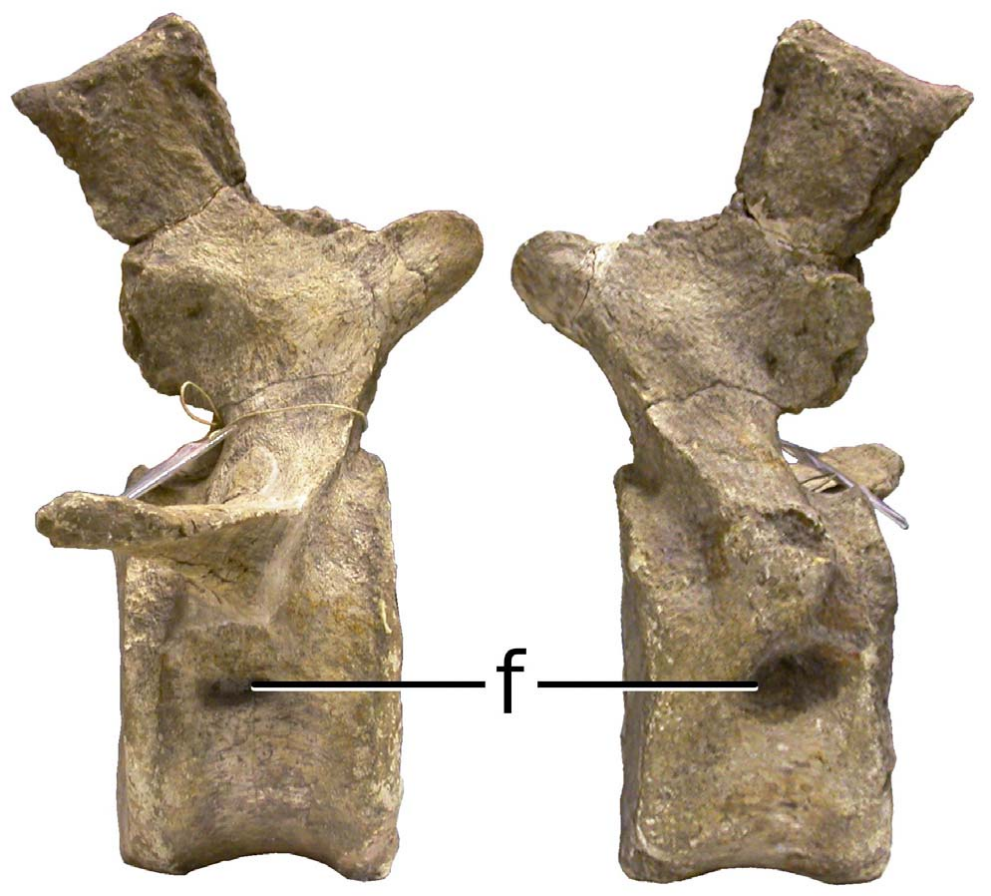
Pneumatic fossae are present only in the second caudal vertebra in several specimens of *Giraffatitan*. Caudal vertebra 2 from the MB.R.2921 (‘Fund Aa’) is shown here in right lateral (left) and left lateral (right) views. Small pneumatic fossae (f) are present on both sides of the centrum, but absent in the rest of the tail. The same pattern of pneumaticity is present in MB.R.3736 (‘Fund D’) and, according to Janensch [57], in the caudal series from the ‘Fund G1’ quarry.

#### MB.R.3736 (‘Fund D’)

MB.R.3736 (‘Fund D’) includes 31 caudal vertebrae, of which caudals 1–23 were found in articulation, with the rest associated. According to Janensch ([57] p. 63), “As in Aa [MB.R.2921], a short and narrow cavity is present below the transverse process of only the second vertebra.” We confirmed that fossae are present on both sides of the centrum in caudal 2 but absent in caudals 1 and 3. This specimen therefore also contains a pneumatic hiatus.

#### Caudal vertebrae from the Gl quarry

Janensch ([57]: p. 66) reported: “The site Gl in the Middle Saurian Marl has yielded weathered remains of *Brachiosaurus* [ =  *Giraffatitan*], portions of extremity bones, and centra from various regions of the tail. Among 15 complete and 6 half centra, one (Gl 4), with ample 25-cm-high posterior end surfaces, distinguishes itself as the second caudal vertebra by its extraordinarily wide ventral surface. It possesses, in accordance with tails Aa and D [MB.R.2921 and 3736], a small lateral depression that is, however, much more clearly formed.” We were unable to locate this vertebra but the distribution of pneumaticity described by Janensch is consistent with MB.R.2921 (‘Fund Aa’) and MB.R.3736 (‘Fund D’).

### Summary of caudal pneumaticity in *Giraffatitan*

#### Patterns of PSP along the tail

**Figure 8:**
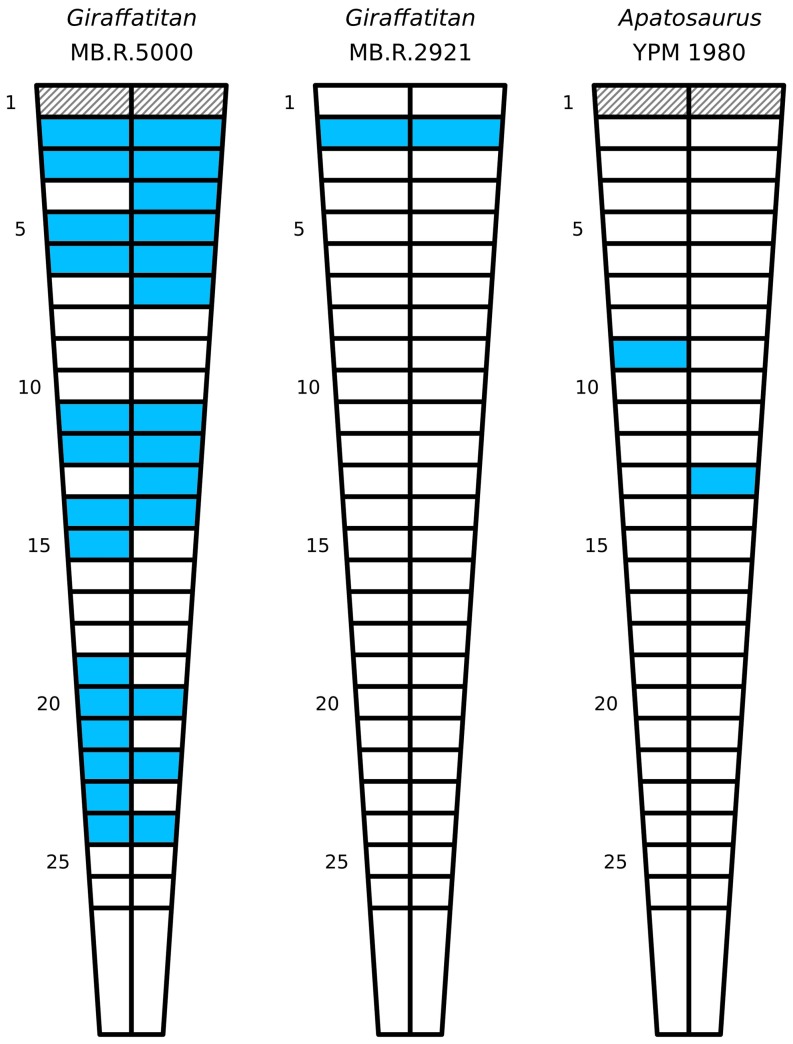
Patterns of caudal pneumaticity in *Giraffatitan* and *Apatosaurus* are complex and frequently include pneumatic hiatuses. Shading conventions follow Figure 4. The intermittent unilateral and bilateral pneumatic hiatuses (i.e., gaps in pneumatization) in *Giraffatitan* MB.R.5000 (‘Fund no’) contrast sharply with the very restricted pneumaticity in MB.R.2921 (‘Fund Aa’) and the isolated pneumatic features in *Apatosaurus* YPM 1980. YPM 1980 has the longest pneumatic hiatuses, unilaterally and bilaterally, that we have found to date in any dinosaur.

The pattern of pneumatization along the MB.R.5000 (‘Fund no’) tail is more complex than in any other known dinosaur (**Figure 8**). PSP varies serially along the tail, from the left to the right side in many of the vertebrae, between the centra and neural arches, and in complex combinations of all three parameters. Proceeding serially from the first preserved vertebrae (caudal 2), there is a block of six pneumatic vertebrae, followed by a bilateral pneumatic hiatus of three vertebrae, then a block of five pneumatic vertebrae, then a second bilateral pneumatic hiatus of three vertebrae, a final block of six pneumatic vertebrae, and finally the apneumatic remainder of the tail. Caudals 2–24 may be considered the total pneumatic domain of the tail, in which skeletal pneumaticity is often but not always present. Asymmetrically pneumatic vertebrae in the anterior half of the domain are apneumatic on the left but never on the right, whereas in the posterior half they are apneumatic on the right but never on the left. The last vertebra that is pneumatic only on the right is caudal 13, and the first vertebra that is pneumatic only on the left is caudal 15, so the switch between these two regions of asymmetric pneumatization occurs in the middle of the second block of pneumatic vertebrae rather than at one of the pneumatic hiatuses.

The *a priori* expectation based on caudal pneumatization in diplodocids [48]–[50], [65] is that PSP would be best developed in the anterior caudals and pneumatic features would diminish monotonically in successively posterior vertebrae. However, this is not the case in MB.R.5000 (‘Fund no’). Except for a fossa in caudal 22 that encroaches on the right side of the neural arch, pneumaticity of the neural elements is found only in four adjacent vertebrae (caudals 11–14) in the second pneumatic block. Furthermore, fossae on the lateral sides of the centra are best developed in the most posterior pneumatic block, caudals 19–24.

The combination of an apneumatic first caudal and pneumatic second caudal is found in at least two specimens, MB.R.2921 (‘Fund Aa’) and MB.R.3736 (‘Fund D’). Janensch described a similar pattern in the vertebrae from the G1 quarry [57], although we were unable to relocate the presumed second caudal with the pneumatic fossae. Although the first caudal of MB.R.5000 (‘Fund no’) is missing, the preserved material is consistent with the same pattern. It will be interesting to see if this pattern holds as the skeletons of more brachiosaurs are discovered in the future.

The differing extent of caudal pneumatization between MB.R.5000 (‘Fund no’) on one hand and MB.R.2921 (‘Fund Aa’) and MB.R.3736 (‘Fund D’) on the other is striking. With so few samples, the cause of the difference is unclear; it could represent ontogenetic or phylogenetic changes or intraspecific variation. MB.R.5000 (‘Fund no’) represents a slightly larger individual than either of the other specimens, and it might have been more mature. However, it would be unusual to have such a large change in the pneumatic domain so late in ontogeny. Taylor [31], [66] has argued on the basis of Migeod's specimen [67] that there is more than one brachiosaurid taxon present in the Tendaguru Formation. It is possible that the variation in caudal pneumaticity between MB.R.5000 (‘Fund no’) and the other Tendaguru brachiosaur specimens carries a phylogenetic signal. For now, though, we assume that all the Tendaguru brachiosaur tails belong to *Giraffatitan*. Pneumatic diverticula show high levels of intraspecific variation in many clades and in different parts of the body (e.g., [68]–[70]), and the seemingly erratic patterns of PSP discussed here could simply represent variation within a population. At least, intraspecific variation is the closest to a null hypothesis among these alternatives.

#### Comparisons to other sauropods

*Giraffatitan* MB.R.5000 (‘Fund no’) is remarkable in having PSP farther posteriorly in its vertebral column than almost any other known sauropod, out to caudal 24. The only other taxa with PSP so far down the tail are saltasaurine titanosaurs: Cerda *et al* ([20]: fig. 4) illustrate pneumaticity down to caudal 25 in *Saltasaurus*. Furthermore, *Giraffatitan* MB.R.5000 (‘Fund no’) has a much larger proportion of its tail pneumatised than the diplodocines. Janensch ([64]) reconstructed *Giraffatitan* with only 55 caudal vertebrae, whereas diplodocines have long caudal series of up to 80 vertebrae ([24]: p. 204). Diplodocines therefore pneumatised only the anterior one quarter of the caudal vertebrae, whereas in *Giraffatitan* PSP is found almost halfway down the caudal series. The situation in saltasaurines is unclear; although rod-like distal caudals were present in some saltasaurines [71], none have been found associated with the same skeletons that preserve extensive caudal pneumaticity. Cerda *et al* ([20]: fig. 4) illustrate between 40 and 50 caudal vertebrae in *Saltasaurus*, in which case PSP was present in 50–60% of the caudal vertebrae.

That Janensch did not mention the numerous pneumatic features in MB.R.5000 (‘Fund no’) is puzzling, given his extensive discussions of PSP elsewhere [57], [72]. From his writing he seems to have considered the anterior and middle caudal vertebrae to be best represented by MB.R.2921 (‘Fund Aa’) and MB.R.3736 (‘Fund D’), respectively, and he valued MB.R.5000 (‘Fund no’) mainly as a source of information about the morphology of distal caudal vertebrae, which were not preserved in the other specimens and which lack pneumatic fossae.

### Caudal pneumaticity in *Apatosaurus*

Although the caudal vertebrae of *Apatosaurus* have been scored as lacking pneumatic fossae or foramina in phylogenetic analyses (e.g., [41]: character 119; [42]: character 181; [73]: character 170), caudal pneumatic features have been documented in the literature for several specimens.

In his description of the “*Brontosaurus*” (now *Apatosaurus*) *excelsus* holotype YPM 1980, the earliest adequate description of any *Apatosaurus* material, Marsh ([52]: p. 417) wrote that “the first three caudals are lightened by excavations in their sides”, and expanded on this saying that “the three vertebrae next behind the sacrum [meaning caudals 1–3] have moderate sized cavities between the base of the neural arch and the transverse processes. These shallow pockets extend into the base of the processes” ([52]: p. 420).

**Figure 9:**
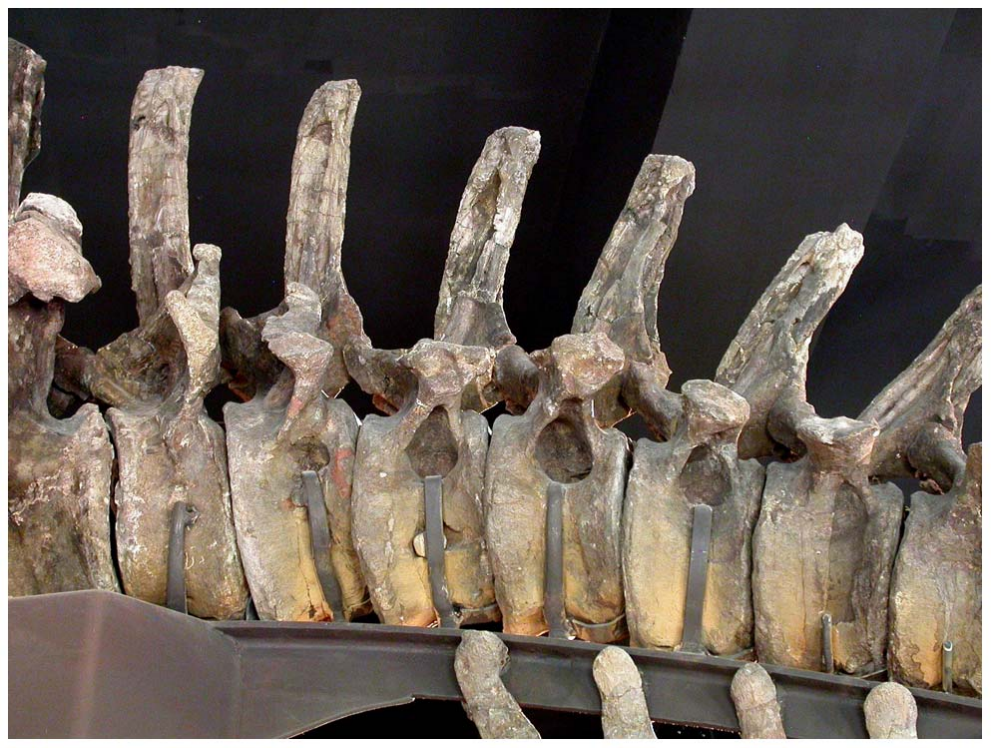
Pneumatic fossae are present in the proximal caudal vertebrae in many specimens of *Apatosaurus*. Here the first part of the tail of FMNH P25112, the mounted *Apatosaurus* skeleton in Chicago, is shown in left lateral view.

Riggs ([53]: p. 188) observed of AMNH 460 that “the number of anterior [caudal] vertebrae having lateral cavities in the centra is five in the Museum specimen” and noted that in the first caudal of his own specimen FMNH P25112 “the interior of the centrum contains numerous small cavities, the pedicles are hollow […] the prezygapophyses […] are excavated at their bases by deep lateral fossae”. He further observed that in the first caudal, “two sets of cavities occur in the centra of the anterior caudal vertebrae, the first above and the second below […] the root of the caudal rib. […] The lateral cavities in the centra persist as far back as caudal V in this specimen” ([53]: p. 189). We have confirmed these observations (**Figure 9**). Riggs ([53]: p. 189) was also first to note the unpredictable distribution of pneumatic features in the tail: “these cavities cannot be regarded as constant characteristics, as they are sometimes present on one side and absent on the other.”

AMNH 222 includes some dorsal, sacral, and caudal vertebrae, originally considered to belong to *Camarasaurus* [74] but since 1900 universally regarded as pertaining to *Apatosaurus*, and in fact incorporated into the mounted skeleton of *Apatosaurus* at the AMNH ([75]: 70; [76]: 375). The proximal caudal vertebrae have complex pneumatic fossae on the neural spines ([74]: fig. 5) and transverse processes ([74]: figs. 3 and 4), and the third caudal vertebra has a prominent pneumatic fossa on the left side of the centrum ([74]: fig. 5).

Gilmore ([24]: p. 203–209), in his detailed discussion of the caudal vertebrae of the *Apatosaurus louisae* holotype CM 3018, surprisingly did not describe any pneumatic features. However, our personal observations show that pneumatic fossae are present on the first three caudals.

Upchurch *et al* [77] reported no caudal pneumaticity in *Apatosaurus ajax* NMST-PV 20375, and wrote, “All caudal centra are solid with no lateral depressions or pleurocoels” ([77]: p. 42). Shallow lateral depressions are illustrated in the anterior caudals ([77]: pl. 5), but these may represent waisting of the vertebrae rather than pneumatic invasion of the bone (see [32]: pp. 212–213 for further discussion of waisting versus pneumatization).

#### YPM 1980

**Figure 10:**
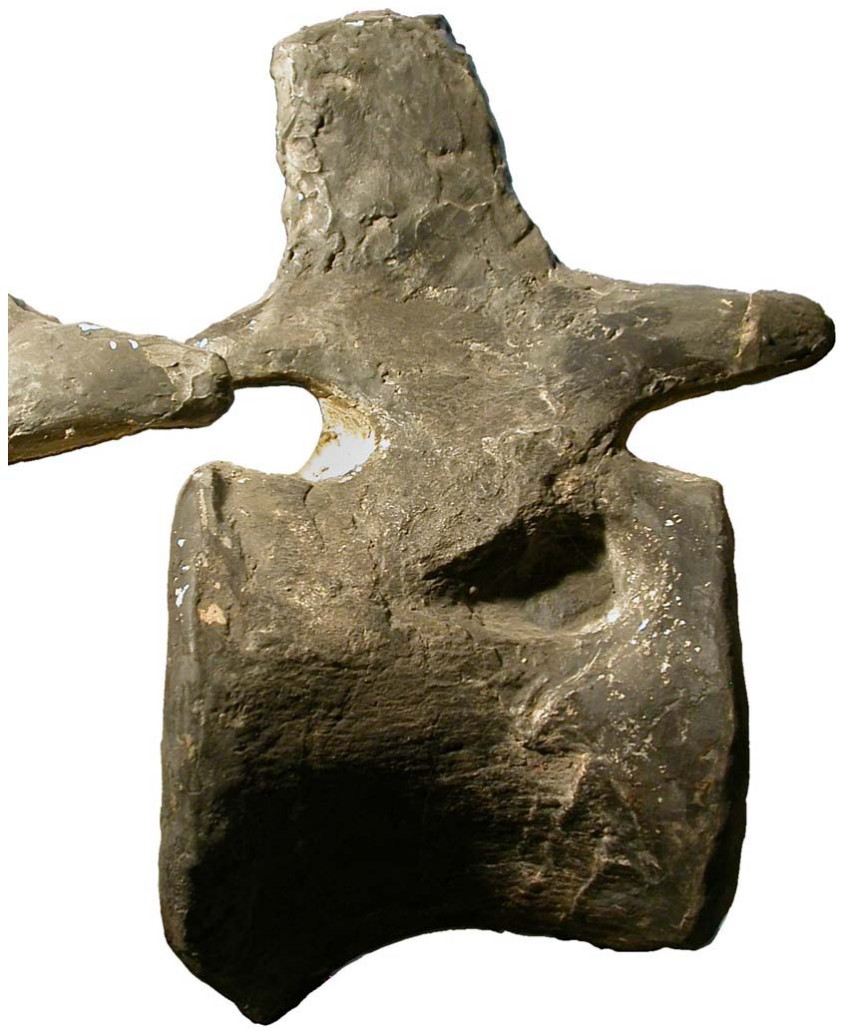
An isolated pneumatic fossa is present on the right side of caudal vertebra 13 in *Apatosaurus excelsus* holotype YPM 1980. The front of the vertebra and the fossa are reconstructed, but enough of the original fossil is visible to show that the feature is genuine.

In our own examination of the mounted *Apatosaurus excelsus* skeleton YPM 1980, we have been unable to locate the lateral excavations described by Marsh. This is surprising because, although many elements of this skeleton were over-enthusiastically “restored” with plaster, obscuring genuine osteological features, the caudal centra after the first are an exception to this, and the bone of the vertebrae, particularly on the right side, is in good condition. The centra of the first dozen or so caudals do feature irregularly positioned lateral foramina (pers. obs., [76]: plates 33–35), but these are very small – less than 1 cm in diameter – and are almost certainly neurovascular rather than pneumatic. It seems unlikely that Marsh was referring to these, especially as they persist long after the first three caudals, but no other features of the bone can be interpreted as matching his description. Much more convincing, however, are two isolated lateral fossae: one on the left side of caudal 9, the other on the right side of caudal 13 (**Figure 10**). Both of these are much larger than the aforementioned foramina – about 6 cm across – and have distinct lips. There is absolutely no trace of similar fossae in any of the other caudals, so these fossae represent a bilateral pneumatic hiatus of at least seven vertebrae (since caudal 1 is extensively reconstructed and may have had pneumatic fossae that cannot be observed) and a unilateral hiatus (on the right side) of at least eleven vertebrae.

### Implications for the development of PSP and its recognition in fossil taxa

Two characteristics of the caudal pneumaticity in *Giraffatitan* and *Apatosaurus* deserve special comment. The first is that the development of pneumatic fossae varies strongly among individuals. MB.R.5000 (‘Fund no’) has numerous distinct, multipartite fossae scattered on the anterior and middle caudal vertebrae, whereas in MB.R.2921 (‘Fund Aa’), MB.R.3736 (‘Fund D’), and the vertebrae from the G1 quarry, caudal pneumaticity is limited to small fossae on the lateral faces of the second caudal centrum. Similarly, YPM 1980 has pneumatic fossae much farther down the tail than in any other known specimen of *Apatosaurus*. The variability of pneumatic traces within the single individuals *Giraffatitan* MB.R.5000 (‘Fund no’) and *Apatosaurus* YPM 1980 is also surprising. PSP is not expressed consistently down the tail, and vertebrae with pneumatic fossae are separated by blocks of vertebrae with no traces of pneumaticity. This inter- and intra-individual variation has several important implications:

#### Pneumatic diverticula were more widespread than their skeletal traces directly indicate

This is not a new insight: in extant birds pneumatic diverticula pass under the skin, in between the muscles, and among the viscera), and only a few of these diverticula leave traces on the skeleton [78]. But it presents a particular problem for paleobiologists because in most cases skeletal evidence is all that we have to work with. Pneumatic hiatuses are present in several articulated caudal series of *Giraffatitan*. The apneumatic first caudal vertebrae of MB.R.2921 (‘Fund Aa’) and MB.R.3736 (‘Fund D’) represent pneumatic hiatuses of one vertebra each, similar to the pneumatic hiatus in the fifth sacral of *Haplocanthosaurus* CM 879 [12]. In MB.R.5000 (‘Fund no’) the pneumatic caudal vertebrae are interrupted by two bilateral pneumatic hiatuses each of three vertebrae. The tail of *Apatosaurus* YPM 1980 has the longest pneumatic hiatus we have found to date—at least seven vertebrae bilaterally, and at least eleven vertebrae unilaterally. Presumably the tails of these sauropods were pneumatized by diverticula of abdominal air sacs which spread distally along the tail during development. Caudal pneumatic hiatuses show that pneumatic diverticula are capable of “leapfrogging” over single vertebrae and even sequences of multiple vertebrae without leaving any diagnostic skeletal traces.

As mentioned above, pneumatic diverticula that leave no traces on the skeleton are common in birds. Within non-avian ornithodirans, pneumatization of distal forelimb elements in pterosaurs suggests the presence of a system of subcutaneous diverticula [7]. We refer to diverticula that do not leave diagnostic skeletal traces as ‘cryptic’ diverticula. The presence of long pneumatic hiatuses in *Giraffatitan* and *Apatosaurus*, the evidence for subcutaneous diverticula in pterosaurs, and the numerous non-skeletal diverticula of birds suggest that cryptic diverticula are a general feature of ornithodiran respiratory systems. Therefore skeletal traces of pneumaticity provide only a lower bound on the extent of the diverticular system, which is often much more extensive and complex in extant birds, and may have been equally extensive and complex in extinct ornithodirans.

#### Asymmetry of inference

Pneumatization of a single element is enough to establish the presence of pneumatic diverticula in a particular region of the body, but even a long string of apneumatic elements does not necessarily indicate that diverticula are absent – as seen with the seven-vertebra bilateral hiatus in the tail of *Apatosaurus* YPM 1980. This asymmetry of evidence and inference is particularly troubling in the case of caudal pneumaticity. As the number of specimens of a taxon without caudal pneumaticity mounts, the likelihood that caudal pneumaticity is absent in the taxon increases, but it can never be truly ruled out because only a single counterexample is needed to demonstrate its presence. The absence of caudal pneumaticity in the many well-described specimens of *Camarasaurus* probably represents a genuine absence (see, e.g., [54]). The same cannot be said for *Brachiosaurus altithorax*, for which the only known caudal vertebrae are the two most anterior caudals of the holotype individual. As *Giraffatitan* demonstrates, *Brachiosaurus* could have invasive caudal pneumaticity that was expressed farther down the tail or in another individual. This seems particularly possible given that Riggs ([21]: p. 235) described a pneumatic hiatus in the sacrum of the *Brachiosaurus* holotype FMNH P25107, in which pneumatic cavities are apparently absent from the second sacral vertebra but present in the first, third and fourth (we have been unable to confirm the presence of this hiatus because the size and fragility of the specimen prevent close examination of the sacral centra).

#### Pneumatic hiatuses do not always indicate separate sources of pneumatization

Pneumatic hiatuses (*sensu* [11]) are less informative than previously supposed. In birds, the only sources of vertebral diverticula posterior to the middle of the dorsal series are the abdominal air sacs, and this was probably true for non-avian saurischians as well ([13], [14], contra [79], [80]). The caudal vertebral diverticula of *Giraffatitan* are therefore inferred to have originated from abdominal air sacs. However, the tail of MB.R.5000 (‘Fund no’) shows that the caudal vertebral diverticula were able to leapfrog over sequences of several vertebrae without leaving any distinct or diagnostic traces, so pneumatic hiatuses do not always indicate that the vertebrae before and behind them were pneumatised by different sources of diverticula. This possibility was recognised by Wedel ([12]: p. 619), but its likelihood was underestimated. The utility of pneumatic hiatuses in determining which air-sacs were the sources of pneumatising diverticula is further undermined by the observation that in juvenile chickens, the middle cervical vertebrae are the first to be completely pneumatised ([12]: fig. 3; [81]). This pneumatization is by diverticula of the cervical air-sacs, and those diverticula leave no osteological traces on the more posterior cervicals that they are also adjacent to: in effect the posterior part of the neck is a cervicodorsal pneumatic hiatus (*sensu* [12]). The same was presumably true in *Pantydraco*, which probably also had pneumatic middle cervicals [32], [82].

This does not mean that pneumatic hiatuses are never produced by multiple sources of diverticula: some of the pneumatic hiatuses of chickens certainly are. (Compare patterns of vertebral pneumatisation in [68]: fig. 1 with mapping of pneumatization domains to air sacs reported by [13], [14]; also see pp. 8-9 and figure 4 in [12].) However, there is currently no way to distinguish hiatuses produced by multiple sources of diverticula from those produced by leapfrogging diverticula, as in *Giraffatitan* and *Apatosaurus*.

#### Pneumatization through ontogeny

It may not be safe to assume that pneumatization of the postcranial skeleton in sauropods is completed in early ontogeny, as it is in the few extant birds in which it has been studied [81], [83]. The restriction of PSP to the second caudal vertebra in all *Giraffatitan* specimens other than MB.R.5000 (‘Fund no’) – assuming they really are all *Giraffatitan*, and not another, as-yet unrecognised taxon – implies that pneumatization of the rest of the tail may have progressed piecemeal throughout ontogeny, and there is no reason to assume that the mounted tail represents the culmination of caudal pneumatization. It is likely that this animal was about the same size as the one represented by MB.R.2181 (HMN SII), from which most of the rest of the mounted skeleton is drawn ([64]: p. 98). However, MB.R.2181 (HMN SII) was probably not fully mature when it died: the suture between the scapula and coracoid is still open, and the individual represented by the fibula MB.R.2688 (HMN XV2) is about 13% larger in linear dimensions. It is possible that fully mature individuals of *Giraffatitan* might have caudal pneumaticity as continuous and invasive as that of diplodocines but extending further down the tail.

#### Morphogenetic rules of postcranial pneumatization

Benson *et al* ([15]: p. 180) identified two morphogenetic rules that appear to govern posterior dorsal and sacral pneumaticity in non-avian theropods. The first is the “neural arch first” rule for posterior expansions of pneumaticity beyond the anterior dorsals. In posterior dorsal and sacral vertebrae of non-avian theropods, if pneumaticity is present, it is always present in the neural arches. The centra may also be pneumatic, but only alongside the arches; one never finds a pneumatic centrum and an apneumatic arch. This is contrast to the “centrum-first” pattern of pneumatic invasion in the cervical vertebrae.

It is not clear if the “neural arch first rule” applies to caudal vertebrae in theropods; Benson *et al* [15] only discussed this rule in the context of dorsal and sacral vertebrae. Using character optimization, Fanti *et al* [27] found that the “neural arch first” rule held for caudal pneumatization in rebbachisaurid sauropods. They interpreted the rule as also applying to theropod caudal vertebrae, and on that basis they proposed that the “neural arch first” pneumatization pattern was synapomorphic for Saurischia ([27]: p. 6).

The second morphogenetic pattern identified by Benson *et al* [15] is the “no gaps” rule, which simply means that there are no gaps in the pneumatization of the vertebral column. The most anterior and posterior pneumatic vertebrae in the entire vertebral column are connected by an unbroken chain of pneumatic vertebrae.

As we discuss above, caudal pneumaticity in *Giraffatitan* and *Apatosaurus* breaks both the “neural arch first” and “no gaps” rules. Regarding the “neural arch first” rule, fossae are occasionally present on the centra but absent on the neural arches in *Giraffatitan* (e.g., the second caudal vertebrae of MB.R.2921 and MB.R.3736, and proximal caudals of MB.R.5000) and *Apatosaurus* (e.g., caudals 9 and 13 of YPM 1980). The same is true of the most distal pneumatic vertebrae in *Diplodocus* (e.g., caudal 18 in AMNH 223, [48]: fig. 13, and caudals 15–19 in USNM 10865, [65]: fig. 3). The situation in some of the mid-caudals in *Giraffatitan* MB.R.5000 is less clear, since the fossae straddle the base of the neural arch and the dorsal part of the lateral centrum. As it stands, “neural arch first” pneumatization of caudal appears to hold in rebbachisaurids [27] but not diplodocines or brachiosaurids, and its status in theropods is unclear. Fanti *et al* [27] proposed “neural arch first” caudal pneumatization as a synapomorphy of Saurischia but that is not supported by this work. Even determining which pattern (“arch first” or “centrum first”) dominates in Sauropoda will require more work.

The “no gaps” rule proposed for non-avian theropods by Benson *et al* [15] does not hold for sauropods. The pneumatic hiatuses described above in both *Giraffatitan* and *Apatosaurus* break this rule, as do those previously described in *Haplocanthosaurus* [12] and *Brachiosaurus* ([21]: p. 235). A pneumatic hiatus may also be present in the basal sauropod *Tazoudasaurus* and in several other basal sauropodomorphs and basal sauropods ([17]: p. 95 and fig. 12). What is most interesting about this apparent pattern is that the very thorough survey of Benson *et al* [15] found no exceptions to the “no gaps” rule among non-avian theropods, but pneumatic hiatuses are present in sauropods and birds [12], which bracket non-avian theropods both phylogenetically and in terms of body size. Clearly more comparative work is needed to elucidate the evolutionary, ecological, and developmental drivers of skeletal pneumatization across Archosauria—the analyses of O'Connor [6], [29], Benson *et al* [15], and Smith [3] are welcome advances, but there are plenty of mysteries left to solve.

### Functional Implications

In the specimens of *Giraffatitan* and *Apatosaurus* discussed herein, PSP does not invade the caudal vertebrae to a significant extent. Reduction of the mass of the vertebrae by pneumatization would have been negligible, a characteristic shared with PSP in early saurischians like *Coelophysis* and *Pantydraco* [32]. This is in sharp contrast to the presacral and sacral vertebrae in *Giraffatitan* and *Apatosaurus*, which were more than 60% air by volume and as lightly built, on average, as the pneumatic long bones of birds [4], [8].

The first postcranial bones to be pneumatised, both ontogenetically in birds and evolutionarily in saurischians, are vertebrae that are not adjacent to the lungs or air sacs, implying that diverticula evolved, and develop, before they interact with the skeleton ([12]: fig. 3; [32]: text-fig. 2). Furthermore, many of the diverticula of extant birds do not pneumatize the skeleton at any point in ontogeny (i.e., all visceral and most intermuscular and subcutaneous diverticula; [78]). These observations suggest that pneumatic diverticula did not evolve to pneumatize the skeleton. (Numerous other possible functions for diverticula are reviewed by Witmer [84].) The very limited resorption of bone during pneumatization in basal saurischians further implies that neither did PSP initially evolve to lighten the skeleton, but it was later exapted for that purpose in lineages where weight loss was important due to great size (sauropods) or flight (birds). Now we find that even in *Giraffatitan* and *Apatosaurus*, both large neosauropods with extensive pneumatization of the presacral and sacral vertebrae, caudal pneumaticity contributed very little to lightening the skeleton. The model of diverticula as “opportunistic pneumatizing machines” ([84]: p. 64) is consistent with many aspects of the development and evolution of skeletal pneumaticity in amniotes. However, it does not explain why presacral and sacral pneumatization in *Giraffatitan* and *Apatosaurus* is so aggressive, whereas caudal pneumatization in the same taxa and the same individuals is so minimal and erratic. This is particularly surprising in light of the fact that, while the torso's mass is suspended between the fore- and hind-limb girdles, the tail is cantilevered, and so its mass induces a large bending moment. It is unlikely that mechanical demands would permit extensive pneumatization of the long, cantilevered neck but prevent pneumatization of the similarly cantilevered tail, which in *Giraffatitan* accounted for only about a third as much volume as the neck ([31]: table 4). The tail of *Apatosaurus* was proportionally much larger, but extensive pneumatization of the tail in the closely related diplodocines (*Diplodocus*, *Barosaurus*, and *Tornieria*), which also had proportionally large tails, suggests that mechanical factors alone are insufficient to explain the very limited caudal pneumatization in *Apatosaurus*.

We hypothesize that in its earliest evolutionary stages, in any part of the body and in any taxon, skeletal pneumaticity has no selective value. In those early stages it confers no disadvantages but does not affect the skeleton enough, through lightening or remodeling individual bones, to offer a selective advantage. It may therefore be invisible to natural selection and free to evolve neutrally (*sensu* [85]). Skeletal pneumaticity can only be favored in those cases where, by chance, it lightens the skeleton enough to become visible to selection. The very limited mass reduction from caudal pneumatization in *Giraffatitan* and *Apatosaurus* suggests that this process of neutral evolution eventually leading, in some cases, to extensive and exaptive skeletal remodeling took place repeatedly in different parts of the body in sauropods. An alternative possibility is that caudal pneumatization was limited by some as-yet-unknown aspect of the developmental program. Cranial skeletal pneumaticity is widespread in extant mammals and archosaurs, and PSP in birds, but the levels of control of the pneumatization process are poorly known. Therefore, neither of these hypotheses can be falsified on the basis of current knowledge, but both could conceivably be tested in extant animals.

## Conclusions

Although it has not been previously recognised, caudal pneumaticity was present in *Apatosaurus* and *Giraffatitan*. Pneumatic fossae in the mid-caudal vertebrae of these animals were not detected for decades following their initial descriptions, despite the fact that two of the most important specimens were on display for most of the twentieth century. Furthermore, the pattern of caudal pneumatization in both taxa appears to have been erratic, although this may be at least partly caused by incomplete ontogenetic sampling. Taken together, these facts suggest that caudal pneumaticity, or at least the capacity to develop it, may be more widely distributed in sauropods (and possibly theropods) than is currently appreciated. We predict that more examples of caudal pneumaticity in otherwise well-known taxa will be discovered in the future.

The discovery of long pneumatic hiatuses in the tails of *Giraffatitan* and *Apatosaurus* complicates our understanding of the development and evolution of PSP in extinct archosaurs, and undermines the utility of hiatuses for identifying the air-sac systems responsible for pneumatization. On one hand, the presence of multiple pneumatic hiatuses within the inferred domain of a single pair of air sacs shows that such hiatuses can be produced by leapfrogging diverticula and do not always indicate pneumatization from multiple sources as originally proposed by Wedel [11]. The pneumatic hiatus reported in *Haplocanthosaurus* [12] seems likely to have been produced by diverticula that simply affected adjacent vertebrae inconsistently. If more pneumatic hiatuses are discovered in extinct ornithodirans, criteria will be needed to distinguish those caused by multiple sources of diverticula from those caused by “leapfrogging” diverticula. Until such criteria are established, the inference that pneumatic hiatuses always indicate multiple air sacs is falsified. However, the case for an essentially avian air sac system in pterosaurs and saurischians is also based on several other lines of evidence [7], [12], and remains robust.

The other major implication of the pneumatic hiatuses in *Giraffatitan* and *Apatosaurus* is that pneumatic diverticula were even more widespread in sauropods than previously thought. This should not be surprising, given the many visceral, intermuscular, and subcutaneous diverticula of extant birds that leave no skeletal traces. The anatomical breadth of diverticular systems in saurischians and pterosaurs is also underscored by distal forelimb pneumaticity in pterosaurs [7].

A common discovery pattern for PSP in pterosaurs and saurischians has been emerging over the past few years: the more we look, the more we find. Compelling evidence of PSP is now known in early representatives of both clades, and patterns of pneumatization in derived pterosaurs, sauropods, and non-avian theropods are diagnostic for the air sacs required for flow-through lung ventilation [7], [12]–[15]. The discovery of more pneumaticity in pterosaurs, sauropodomorphs, and non-avian theropods emphasises how strange is the absence of reported pneumaticity in ornithischians ([16]: p. 19; the putative pneumatic foramen in a dorsal rib of the iguanodont *Delapparentia* [86] is not convincing). If, as seems increasingly likely, an air sac system is primitive for Ornithodira, why did ornithischians never discover PSP (in a developmental sense)? And if an air sac system is not primitive for Ornithodira, why did the three other major lineages evolve PSP so soon after their divergence from one another and from Ornithischia?

It is possible that ornithischians did have pneumatic diverticula, but that—following the hypothesis of initially neutral evolution described above—these diverticula did not impact the skeleton enough to become visible to selection. This is a complex scenario that will be difficult to test, since we currently have no way of identifying pneumatic diverticula in fossil taxa other than by their skeletal traces. In basal sauropodomorphs, potentially pneumatic fossae can be difficult to assess because the recesses ventral to the diapophyses are often obscured by sediment, even in apparently well-prepared specimens ([16]: p. 16; [17]: 95). Largely because of this difficulty, PSP went unrecognized in basal sauropodomorphs until very recently. By analogy, we think it is at least possible that pneumatic fossae in ornithischians, if present, may have escaped detection. We therefore encourage paleobiologists to keep an eye out for even rudimentary indications of PSP in ornithischians.
